# Identification of Lenalidomide Sensitivity and Resistance Mechanisms in Non-Del(5q) Myelodysplastic Syndromes

**DOI:** 10.3390/ijms21093323

**Published:** 2020-05-08

**Authors:** Leylah M. Drusbosky, Christopher R. Cogle

**Affiliations:** Division of Hematology and Oncology, Department of Medicine, College of Medicine, University of Florida, Gainesville, FL 32610, USA; ldrusbosky@gmail.com

**Keywords:** myelodysplastic syndromes, refractory disease, resistance, computational modeling

## Abstract

Whereas lenalidomide is an effective therapy for del(5q) MDS patients, a minority of non-del(5q) MDS patients achieve hematologic improvement with lenalidomide. We used computational biology modeling and digital drug simulation to examine genomic data from 56 non-del(5q) MDS patients treated with lenalidomide, and then matched treatment response with molecular pathways. The computer inferred genomic abnormalities associating with lenalidomide treatment response in non-del(5q) MDS to include trisomy 8, del(20q), or *RUNX1* loss of function mutations. Genomic abnormalities associating with lenalidomide resistance in non-del(5q) MDS patients included mutations in *SF3B1*, *TET2*, *WNT3A* amplification, *MCL1* amplification, and/or *PSEN2* amplification. These results may inform protocols for determining appropriateness of lenalidomide in non-del(5q) MDS.

## 1. Introduction

**Figure 1:**
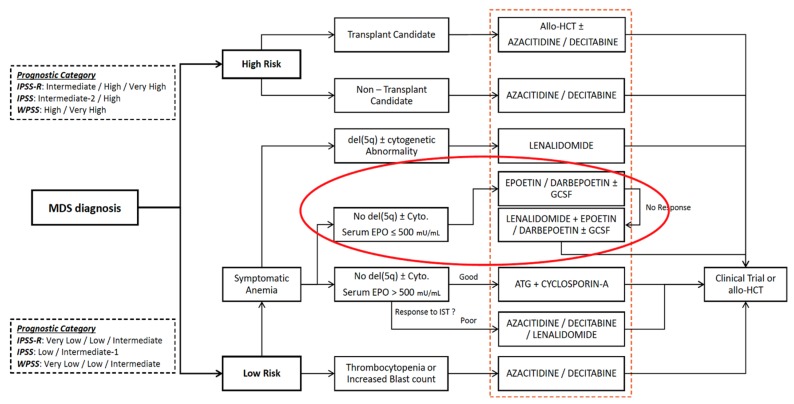
NCCN Guidelines for the Treatment of MDS. The orange dashed box highlights all treatment recommendations for MDS. The red circle highlights treatment options for non-del(5q) MDS.

Low-dose lenalidomide is a highly effective therapy in patients with del(5q) MDS, reducing need for transfusions in 76% of patients and achieving transfusion independence in 67% of patients [1]. However, del(5q) MDS is a rare subtype affecting < 5% of MDS patients. Most patients with MDS have non-del(5q) disease karyotype and only 27% of these patients achieve transfusion independence with low-dose lenalidomide [2]. Although the NCCN has recognized the clinical utility of lenalidomide for non-del(5q) MDS patients after failure of erythropoiesis-stimulating agent (ESA) treatment (Figure 1) [3], unfortunately, there are no biomarkers to gauge the likelihood of treatment response in non-del(5q) MDS patients. Thus, treating non-del(5q) MDS patients with lenalidomide is risky because most patients will not achieve hematologic improvement despite high risk for toxicities. 

Identifying biomarkers for predicting lenalidomide sensitivity or resistance in the non-del(5q) MDS patient population would greatly improve current clinical MDS management by enabling more definitive patient selection or rejection to receive lenalidomide treatment.

A genotyping study in non-del(5q) MDS patients showed persistence of dominant subclones despite lenalidomide treatment, but did not conclusively identify molecular pathways responsible for lenalidomide resistance [4]. Thus, reasons for lenalidomide sensitivity or resistance in non-del(5q) MDS is still an open question. Furthermore, identification of lenalidomide resistance pathways may inform the selection of adjuvant therapies that mitigate resistance mechanisms. Ultimately, rationally designed, new drug combinations could be constructed on a lenalidomide backbone for MDS patients.

Recently, we developed a computational biology modeling (CBM) and digital drug simulation system fed by clinical genomic data from MDS and AML patients that accurately models MDS and treatment response to drugs such as lenalidomide [5,6]. Importantly, our CBM system displays intercellular pathways that drive sensitivity or resistance to drug therapies. In this study, we sought to fill the knowledge gaps with respect to lenalidomide sensitivity or resistance in non-del(5q) MDS by using our CBM system.

## 2. Results

### 2.1. Sufficiency of Genomic Information for Computational Biology Modeling

**Figure 2:**
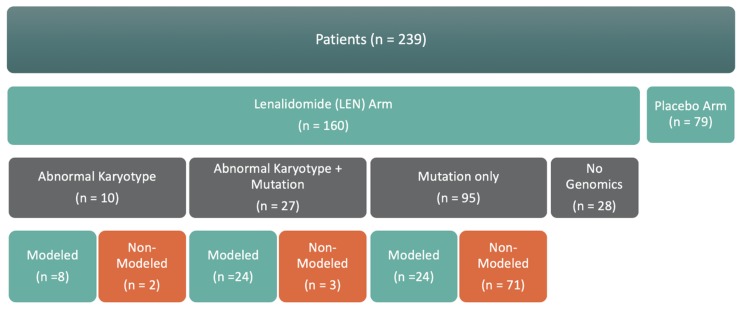
Summary of Genomic Information Sufficiency for Computational Modeling.

From January 2010 to March 2013, 239 patients with transfusion-dependent non-del(5q) MDS were recruited and treated with either lenalidomide (160/239, 67%) or placebo (79/239, 33%). Of the 160 patients receiving lenalidomide treatment, 10 had abnormal karyotype, 27 had abnormal karyotype plus a gene mutation, 95 had a gene mutation and normal karyotype (mutation only), and 28 had no gene mutation detected and normal karyotype (no genomics) (Figure 2). Of the 132 with genomic mutation results, 56 had enough genomic results to produce a computational biology model for digital drug simulations.

### 2.2. Non-Del(5q) MDS with Abnormal Karyotype

**Figure 3:**
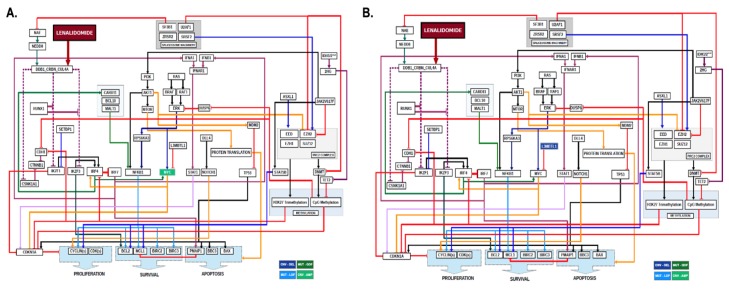
**Computational Models of Non-Del(5q) MDS Patients with Abnormal Karyotype.** MDS patient-specific gene copy number variations derived from karyotype result were computationally modeled and lenalidomide treatment digitally simulated. Two-dimensional maps showing intracellular pathways show the mechanisms of lenalidomide sensitivity in non-del(5q) MDS patients with (**A**) trisomy 8 karyotype and (**B**) del(20q) karyotype. Arrowed lines indicate agonistic relationship. Barred lines indicate antagnostic relationship. Line colorations are to assist the human eye in discriminating among the pathways. Somatic genes in dark blue boxes indicate copy number variation (CNV) deletions (DEL) derived from cytogenetic abnormalities. Somatic genes in light green indicate CNV amplifications (AMP) derived from cytogenetic abnormalities. Somatic genes with mutations resulting in loss of function (LOF) are indicated in light blue boxes. Somatic genes with mutations resulting in gain of function (GOF) are indicated in dark green boxes.

Within the abnormal karyotype group, seven of eight patients modeled achieved a clinical response with lenalidomide and were found by CBM to have a trisomy 8 and/or del(20q) karyotype that were predicted to enhance lenalidomide sensitivity because of increased Myc, either from a *MYC* gene copy number increase (amplification) or a *MYC* repressor gene (L3BMTL1) copy number decrease (deletion) (Figure 3).

### 2.3. Non-Del(5q) MDS with Abnormal Karyotype and Gene Mutations

**Figure 4:**
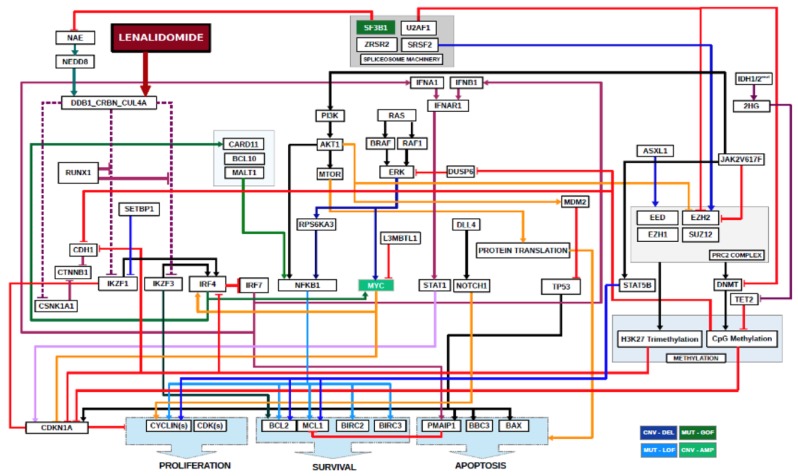
**Computational Models of Non-Del(5q) MDS Patients with Abnormal Karyotype and Genetic Mutations.** MDS patient-specific gene copy number variations, gain-of-function gene mutations, and loss-of-function gene mutations were computationally modeled and lenalidomide treatment digitally simulated. Two-dimensional maps showing intracellular pathways show the mechanisms of lenalidomide resistance in non-del(5q) MDS patients with a trisomy 8 karyotype and a mutation in *SF3B1.* Arrowed lines indicate agonistic relationship. Barred lines indicate antagnostic relationship. Line colorations are to assist the human eye in discriminating among the pathways. Somatic genes in dark blue boxes indicate copy number variation (CNV) deletions (DEL) derived from cytogenetic abnormalities. Somatic genes in light green indicate CNV amplifications (AMP) derived from cytogenetic abnormalities. Somatic genes with mutations resulting in loss of function (LOF) are indicated in light blue boxes. Somatic genes with mutations resulting in gain of function (GOF) are indicated in dark green boxes.

Within the abnormal karyotype and gene mutation group, 19 of 24 patients modeled failed to achieve a clinical response to lenalidomide and were found by CBM to have somatic mutations in *SF3B1*, which were predicted to result in lenalidomide resistance because of enhanced degradation of NEDD8 activating enzyme (NAE) that was predicted to reduce ubiquitin conjugation activity of cereblon—the mechanistic target of lenalidomide (Figure 4). With reduced cereblon activity in the MDS network, it was reasoned that the MDS will be resistant to lenalidomide. Other genomic abnormalities observed in clinically resistant cases included *TET2* mutations through mediation of *CDKN1A*, *JAK2* V617F mutation due to activation of *STAT5B*, and chromosome 1q amplification due to gene dosage increases in *WNT3A*, *MCL1*, and *PSEN2*.

### 2.4. Non-Del(5q) MDS with Normal Karyotype and Gene Mutations

**Figure 5:**
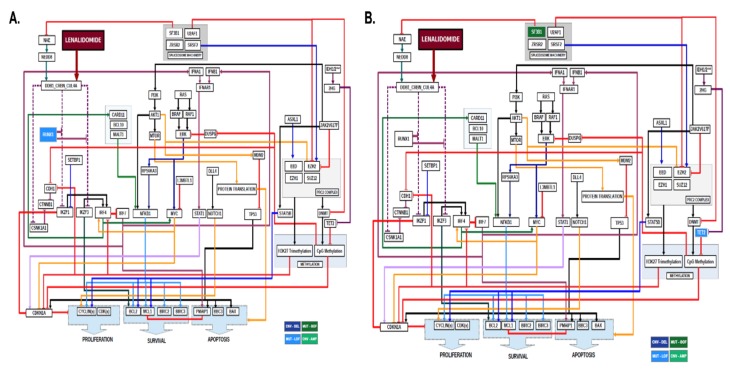
**Computational Models of Non-Del(5q) MDS Patients with Genetic Mutations and Uninformative Karyotype.** MDS patient-specific gene copy number variations, gain-of-function gene mutations, and loss-of-function gene mutations were computationally modeled and lenalidomide treatment digitally simulated. Two-dimensional maps showing intracellular pathways show the mechanisms of lenalidomide sensitivity in (**A**) *RUNX1* mutant MDS. (**B**) Lenalidomide resistance was observed in non-del(5q) MDS cases harboring mutations in *SF3B1* and *TET2.* Arrowed lines indicate agonistic relationship. Barred lines indicate antagnostic relationship. Line colorations are to assist the human eye in discriminating among the pathways. Somatic genes in dark blue boxes indicate copy number variation (CNV) deletions (DEL) derived from cytogenetic abnormalities. Somatic genes in light green indicate CNV amplifications (AMP) derived from cytogenetic abnormalities. Somatic genes with mutations resulting in loss of function (LOF) are indicated in light blue boxes. Somatic genes with mutations resulting in gain of function (GOF) are indicated in dark green boxes.

Within the group of non-del(5q) patients with gene mutations and normal karyotype on cytogenetic testing, 22 of 24 patients modeled failed to achieve a clinical response to lenalidomide and were found by CBM to harbor mutations in *SF3B1* and/or *TET2* (Figure 5).

A summary of gene signatures and biomarkers relative to modeling cohort and treatment response is presented in Table 1.

## 3. Discussion

In this report, we used a computational method to identify recurrent karyotype abnormalities and gene mutations associating with either lenalidomide sensitivity or resistance in non-del(5q) MDS patients.

In general, trisomy 8 or del(20q) was associated with lenalidomide sensitivity putatively through *MYC* amplification or *MYC* repressor (L3MBTL1) deletion. The connections among dysregulated Myc, proteasomal substrate proteins Ikaros (IKZF1) and Aiolos (IKZF3), and lenalidomide have been verified in multiple myeloma [7], but not yet in MDS. In addition to associating with lenalidomide sensitivity, we found that *RUNX1* loss of function mutations were associated with treatment response theoretically due to release of IKZF1/3 repression. In a prior report, RUNX inhibition sensitized multiple myeloma cells to lenalidomide [8], and MDS cells have yet to be tested.

Genomic abnormalities associating with lenalidomide resistance in non-del(5q) MDS patients included mutations in *SF3B1*, *TET2*, *WNT3A* amplification, *MCL1* amplification, and/or *PSEN2* amplification. Our CBM system inferred that the *SF3B1* K700E mutation mediates NAE degradation, which reduces cereblon activity, and therefore lessens response to lenalidomide. This result provides a new hypothesis for follow-up testing. The connection between Wnt/B-catenin pathway activation and lenalidomide resistance has been verified in multiple myeloma [9], but not yet in MDS. There are many pathways to activating the Wnt/B-catenin pathway in MDS, and the one encountered in the patient cohort studied was via an additional chromosome 1q.

This study extends prior efforts to identify biomarkers of lenalidomide treatment response in patients with myeloid malignancies, as reviewed in several earlier publications [10,11,12]. Previous work associating biomarkers with clinical outcomes can be organized into four categories: somatic genomic abnormalities, cereblon target variation, immune related biomarkers, or gene expressions. Some groups have examined lenalidomide treatment response in association with somatic genomic abnormalities and found no link [13]. While other groups have identified unmutated *TP53* associated with hematological improvement in del(5q) MDS patients treated with lenalidomide [14,15]. However, in that study, 16% of del(5q) MDS patients with wild type *TP53* progressed to AML despite lenalidomide treatment, which indicates that there are additional factors besides this single genetic biomarker involved in lenalidomide response. In a separate cohort of del(5q) MDS patients, the presence of DNMT3A was associated with lenalidomide treatment response [16]. In a small number of non-del(5q) MDS patients, somatic mutations in *SF3B1* and *TET2* correlated with nonresponse to lenalidomide [16], which is consistent with the current study. Thus, results from prior efforts are mixed. In the current study, rather than use single biomarker correlation statistics, we used a computational modeling system capable of simultaneously projecting hundreds of genomic abnormalities, such as copy number variations, loss of function gene mutations, and gain of function gene mutations, and then digitally simulating lenalidomide treatment. We believe that the totality of genomic abnormalities is a more accurate representation of the multiple factors affecting lenalidomide treatment response.

In a prior report, a small number of MDS patients with isolated del(5q) showed decrease in *CRBN* expression levels after lenalidomide treatment that also correlated with treatment response [17]. In the current study, we took into account cereblon expression as controlled by upstream regulators and *CRBN* gene copy number variations. However, *CRBN* expression level may not be an important lenalidomide treatment response factor in lower risk non-del(5q) MDS patients [18]. Furthermore, we did not take into account cereblon genetic polymorphisms, which have been identified and proposed as a biomarker of response to lenalidomide treatment in non-del(5q) MDS patients [18].

In this study, we found immune system-related cytokines as important mediators of lenalidomide response to treatment. Likewise, increased serum concentration of the inflammatory protein, S100A9, and decreased serum concentration of its nuclear factor kappa-light-chain-enhancer of activated B cells (NF-κB) transcriptional target, tumor necrosis factor alpha (TNF-α), have been linked to lenalidomide treatment response in non-del(5q) MDS patients [19]. Although the estimated abundance of T cells increased over time in non-del(5q) MDS patients who responded to lenalidomide, no immune cell abundance (T, B, or NK) before treatment predicted lenalidomide response [20]. The current study only evaluated intracellular genomic abnormalities and did not assess the extrinsic abundance of immune cells.

Lastly, agnostic gene expression studies have been performed retrospectively in MDS patients treated with lenalidomide. In a small study of MDS patients treated with lenalidomide, 47 genes were more highly expressed in nonresponders than responders and an additional six genes were more highly expressed in responders than nonresponders [21]. From those 53 genes, the investigators chose to focus on 30 genes involved in erythroid differentiation. It should be noted that, in the prior study, although the training set of eight patients included non-del(5q) MDS, the validation test cohort of 26 patients included 13 (50%) with del(5q) MDS. Thus, it cannot be stated conclusively that the genes identified in the prior study were specific to non-del(5q) MDS.

Several additional considerations should be made when interpreting results from the current study. First, the number of patients in each subgroup are limited. Although only reproducible findings are presented and not single case observations, a larger sample size would increase confidence in associating genomic biomarkers with response. Second, biological assays are needed to verify results. 

After verifications, these results could inform precision hematology-oncology protocols for determining appropriateness of administering lenalidomide treatment to patients with non-del(5q) MDS.

## 4. Methods

### 4.1. Patients

The University of Florida IRB approved this retrospective study by waiver of consent under Title 45 CFR 46.116(f) (Common Rule) (UF IRB #201600284). Patients with transfusion dependent non-del(5q) MDS were prospectively recruited to a phase III randomized clinical trial of lenalidomide versus placebo (NCT01029262, MDS-005) [22]. The trial randomized participants 2:1 to lenalidomide or placebo. The IPSS cytogenetic risk groups from the MDS-005 clinical trial were 82.4% Good Risk, 17.2% Intermediate Risk, and missing in 0.4%. Clinical responses were recorded according to IWG 2006 response criteria [23].

### 4.2. Genomic Testing

Diagnostic bone marrow specimens from the patients were examined by chromosome karyotyping and a 56-gene next-generation sequencing (NGS) panel and variant allele frequency (VAF) cutoff levels were as used in the prior report [22].

### 4.3. Computational Biology Modeling and Digital Drug Simulation System

**Figure 6:**
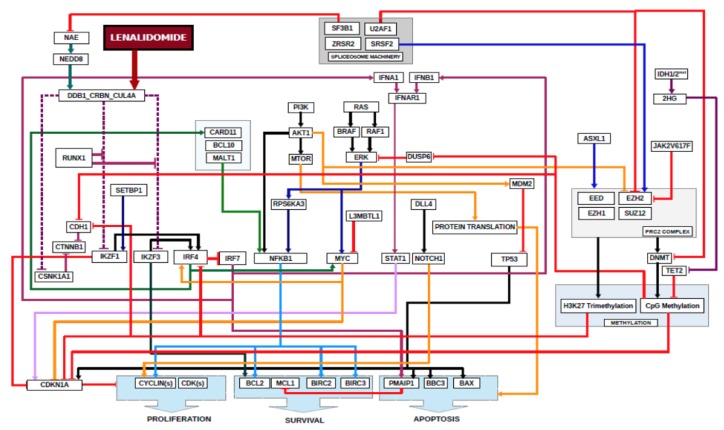
**Schematic of Digital Lenalidomide Drug Model in MDS.** Software code for lenalidomide was written to include its direct target, cereblon, and required downstream mediators that result in decreases in cell proliferation, survival and increased apoptosis. Also depicted are somatic genes frequently mutated in MDS and their relationships to downstream mediators of lenalidomide sensitivity. Arrowed lines indicate agonistic relationship. Barred lines indicate antagnostic relationship. Line colorations are to assist the human eye in discriminating among the pathways.

A computational biology model (CBM) and digital drug simulation system was used to model each patient’s MDS, similar to previous reports [5,6]. A model of lenalidomide was created to include lenalidomide’s target and downstream mediators (Figure 6).

## Figures and Tables

**Table 1:** Summary of Gene Signatures and Biomarkers Associating with Lenalidomide Sensitivity or Resistance.

		Gene Signature		Biomarker	
Groups	Summary	Sensitive	Resistance	Sensitive	Resistance
Abnormal Karyotype	88.89% of patients are Responders to Lenalidomide due to the presence of Trisomy 8 or 20q deletion. Resultant patients would have higher levels of MYC in the system, one of the factors in the sensitivity loop for Lenalidomide.	MYC (AMP), L3BMTL1 (DEL)	-	MYC	-
Abnormal Karyotype and Gene Mutations	81.48% of patients are Non-Responders to Lenalidomide due to the presence of mutations in *SF3B1* in combination with Trisomy 8 and 20q deletion. Also, the presence of 1q amplification in patients who are Non-Responders to Lenalidomide.	MYC (AMP), L3MBTL1 (DEL)	SF3B1-K700E, TET2 (LOF), WNT3A (AMP), MCL1 (AMP), PSEN2 (AMP)	MYC	CTNNB1, NOTCH1, MCL1, SF3B1, TET2
Gene Mutations only	91.67% of patients are Non-Responders to Lenalidomide due to presence of mutations in *SF3B1* and methylation machinery.	RUNX1 (LOF)	SF3B1-K700E, TET2 (LOF)	RUNX1	SF3B1, TET2
All	74.58% of patients were Non-Responders to Lenalidomide.	MYC (AMP), L3MBTL1 (DEL), RUNX1 (LOF)	SF3B1-700E, TET2 (LOF), WNT3A (AMP), MCL1 (AMP), PSEN2 (AMP)	MYC, RUNX1	CTNNB1, NOTCH1, MCL1, SF3B1, TET2
